# Cell-Free Double-Stranded DNA to DNase Ratio Predicts Outcome after Primary Survived Cardiac Arrest

**DOI:** 10.3390/cells11213367

**Published:** 2022-10-25

**Authors:** Richard Rezar, Michael Lichtenauer, Vera Paar, Adrienne Aszlan, Thomas M. Hofbauer, Reinhard Kaufmann, Sarah Wernly, Clemens Seelmaier, Moritz Mirna, Andreas Mangold, Irene M. Lang, Uta C. Hoppe, Anna S. Ondracek, Bernhard Wernly

**Affiliations:** 1 Department of Cardiology and Intensive Care Medicine, Paracelsus Medical University of Salzburg, Müllner Hauptstraße 48, 5020 Salzburg, Austria; 2 Department of Internal Medicine II, Division of Cardiology, Medical University of Vienna, Währinger Gürtel 18-20, 1090 Vienna, Austria; 3 Department of Radiology, Paracelsus Medical University of Salzburg, Müllner Hauptstraße 48, 5020 Salzburg, Austria; 4 Department of Internal Medicine, General Hospital of Oberndorf, Paracelsusstraße 37, 5110 Oberndorf bei Salzburg, Austria; 5 Center for Public Health and Healthcare Research, Paracelsus Medical University of Salzburg, Strubergasse 21, 5020 Salzburg, Austria; 6 Department of Internal Medicine I, Landeskrankenhaus Feldkirch, Carinagasse 47, 6800 Feldkirch, Austria

**Keywords:** double-stranded DNA, deoxyribonuclease, cardiopulmonary resuscitation, post-cardiac arrest syndrome, critical care outcomes

## Abstract

(1) Double-stranded DNA (dsDNA) and deoxyribonuclease (DNase) as surrogate parameters for accumulating inflammatory hazards are insufficiently studied in resuscitation research. (2) Blood samples of 76 individuals after CA were analyzed 24 and 96 h after ICU admission. Plasma levels of dsDNA, interleukin-8, and monocyte chemoattractant protein-1 and activity of DNase were assessed along with baseline characteristics, intensive care measures, and outcome data. DsDNA/DNase ratio was used as main prognostication parameter. After calculating an optimal empirical cut-off for outcome prediction (death or Cerebral Performance Category ≥3 at 6 months), multivariable logistic regression was applied. (3) Using receiver operating characteristic (ROC) analysis, an area under the curve (AUC) of 0.65 (95% CI 0.50–0.79) was found for dsDNA/DNase after 24 h versus 0.83 (95% CI 0.73–0.92) after 96 h (*p* = 0.03). The empirical cut-off for dsDNA/DNase ratio after 96 h was 149.97 (Youden). DsDNA/DNase ratio was associated with unfavorable outcome at six months (aOR 1.006, 95% CI 1.0017–1.0094, *p* = 0.005). In multivariable analysis, the association of dsDNA/DNase ratio independently predicted outcome as a continuous variable (aOR 1.004, 95% CI 1.0004–1.0079, *p* = 0.029) after adjusting for potential confounders. (4) DsDNA/DNase ratio at 96 h demonstrates good predictive performance for estimating outcome after CA.

## 1. Introduction

**Figure 1:**
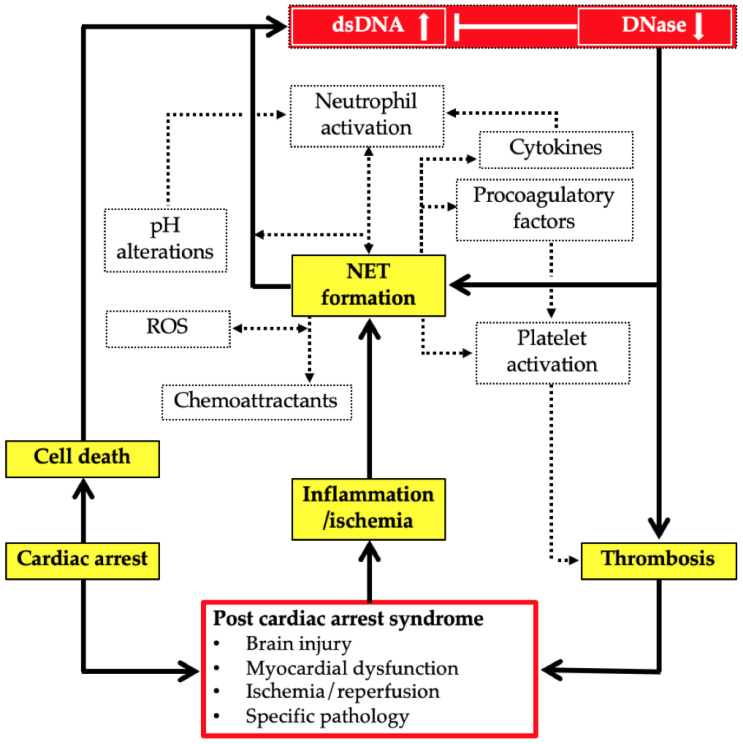
Schematic representation of the potential pathophysiological role of neutrophil extracellular traps (NETs) in post-cardiac arrest syndrome (PCAS). Abbreviations: DNase: deoxyribonuclease; dsDNA double-stranded DNA; NET: neutrophil extracellular traps; PCAS: post cardiac arrest syndrome; ROS: reactive oxygen species.

Primary survived cardiac arrest (CA) is a common condition in critical care, and the proportion of patients discharged alive is small (on average 8%) [1,2]. Despite significant therapeutic advances, it remains difficult to improve outcome after primary restoration of spontaneous circulation (ROSC). In particular, post-cardiac arrest syndrome (PCAS) represents a unique pathophysiological complex. In addition to cerebral injury, myocardial dysfunction, and, depending on the cause of arrest, persistent precipitating pathology, a generalized acute phase response in the setting of systemic ischemia-reperfusion injury is a particularly difficult problem to address [3]. Neutrophil granulocytes are the most abundant white blood cells in humans and part of the innate immune system. They are essential for the primary defense against microorganisms and play a role in the pathophysiology of cardiovascular disease: Upon activation, neutrophils form neutrophil extracellular traps (NETs), which consist of extracellular DNA strands and acute phase proteins subsequently interacting with other blood components. NET formation is influenced by multiple factors, including hypoxia, hypercapnia, pH changes, and circadian factors [4,5]. A graphical overview of the pathophysiological role of NETs in the context of PCAS is shown in Figure 1. The role of the pro-inflammatory chemokines interleukin-8 and monocyte chemotactic protein-1 (MCP-1) has also been studied previously in patients after CA [6,7]. The generalized inflammatory response in the context of regaining circulation after cardiac arrest represents a stress state in the body, which shares similarities with the pathophysiology of sepsis, but on the other hand is unique as an entity due to the short-term complete ischemia and reperfusion injury [8]. DsDNA might not only be a marker of cell death in ischemia-reperfusion injury of CA patients, but per se, and especially as a NET component, may act as an additional pro-inflammatory stimulus eliciting overshooting immune responses [9,10]. Some published evidence also suggests a harmful effect of chronic, low-grade dsDNA burden attributable to a deficient DNase activity [11]. Therefore, several accumulating factors might determine patient outcome in CA represented by the balance of dsDNA and DNase: (1) extensive tissue damage, (2) inflammatory signaling, or (3) a reduced capacity to organize and contain the damage response. In this study, we sought to examine the dsDNA/DNase ratio as a predictor of outcome, subsuming a potentially unbalanced degree of cell death and immunological responses in a cohort comparable to previous studies, yet also including a later time point (96 h after CA) to account for a possibly even longer lasting inflammatory reaction [12,13]. Furthermore, we tried to contribute to a better understanding of the ongoing processes in PCAS by investigating novel inflammatory biomarkers (IL-8, MCP-1) on top. With our study, we attempted to evaluate the role of persistent inflammation and associated poor outcomes after CA.

## 2. Materials and Methods

### 2.1. General Study Course

The study site was the intensive care unit (ICU) of the Department of Cardiology and Intensive Care Medicine (Paracelsus Medical University Salzburg). Patients after cardiopulmonary resuscitation (CPR) with a minimum age of eighteen years who were admitted to the ICU between 12/2018 and 03/2020 were included in this observational, single-center study. Written informed consent was obtained when possible; for deceased patients or individuals with poor neurologic outcome, informed consent was obtained from next of kin when appropriate. Both patients after out-of-hospital cardiac arrest (OHCA) and in-hospital cardiac arrest (IHCA) were included. Exclusion criteria were (1) traumatic cardiac arrest and (2) hospitalization of less than 24 h (the latter due to logistical reasons). A positive ethics vote was previously obtained (415-E/2408/8-2018), and the study was conducted according to the principles of the Declaration of Helsinki and Good Clinical Practice. Standard laboratory tests were performed according to the hospital laboratories standards. Study blood samples were collected 24 and 96 h after hospital admission. For the laboratory analyses, serum tubes were centrifuged in the first twenty minutes after collection. Afterwards the supernatant was stored at −20 °C for a maximum of one month after collection for logistical reasons. To prevent loss of quality, samples were deep-frozen at −80 °C for further storage. Levels of interleukin-8 (IL-8) and monocyte chemotactic protein 1 (MCP-1) were analyzed via commercially available enzyme-linked immunosorbent assay kits (ELISA) (DuoSet ELISA DY208 & DY279 R&D Systems, Minneapolis, MN, USA) analogous to our measurements from the indicated publication [14]. Telephone follow-ups were performed at 30 days and six months and included neurological assessment by Cerebral Performance Category (CPC). The CPC is divided into five scores. CPC 1 represents a good neurological outcome (mild neurological deficit at most), CPC 2 represents a moderate cerebral disability (independent in activities of daily life, work in protected environment possible). A CPC 3 stands for a severe cerebral disability (dependent from others, includes a wide range up to severe dementia or paralysis). CPC 4 represents a coma or vegetative state, CPC 5 brain death. A CPC ≥ 3 is generally considered an unfavorable outcome in resuscitation research and was used accordingly in this work. For comparative purposes, healthy control subjects (*n* = 75) were recruited at the Health and Prevention Center Sanatorium Hera in the course of routine health check-ups. Volunteers were enrolled after giving written informed consent. Medical history and clinical parameters were documented. Blood was drawn for routine laboratory analysis, and measurement of dsDNA levels and DNase activity. The study was approved by the Ethics Committee of the Medical University of Vienna (EK 1947/2014).

### 2.2. Measurement of dsDNA

Circulating cell-free dsDNA was measured using the intercalating agent Sytox Green nucleic acid stain (Thermo Fisher Scientific, Waltham, MA, USA) in a 96-well plate format as previously described [15]. Plasma samples were diluted 1:10 in dilution buffer (0.1% bovine serum albumin (BSA), 5 mmol/L ethylenediaminetetraacetic acid (EDTA) in phosphate buffered saline) and then mixed 1:2 with 2 µM Sytox Green. Lambda DNA (Thermo Fisher Scientific, Waltham, MA, USA) diluted to the highest final concentration of 250 ng/mL served as a standard with an assay detection limit of 3.9 ng/mL. After two minutes of incubation in the dark at room temperature, fluorescence was measured using a Glomax microplate reader (Promega, excitation: 480 nm, emission: 520 nm). Auto-fluorescence was measured by diluting samples 1:4 in dilution buffer without Sytox Green. After subtraction of background and blank values, remaining fluorescence intensities of plasma samples were calculated against the standard curve.

### 2.3. Total DNase Activity

**Figure A1:**
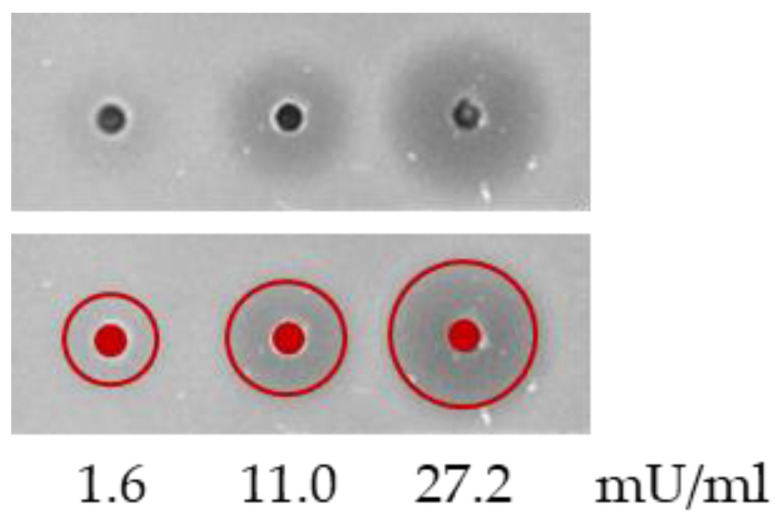
Single radial enzyme diffusion (SRED) assays were analyzed using ImageJ. Per sample, a degradation index was calculated based on circumference (big red circle) and background-corrected intensity of the digested area, excluding the well (filled small circle). Representative SRED assays of three different samples with low, intermediate, and high DNase activity [mU/mL] are shown.

Total DNase activity was measured using a single radial enzyme diffusion (SRED) assay as previously published [7]. An example is shown in Appendix A, Figure A1. Salmon testes DNA serving as substrate for DNases (Sigma-Aldrich, St. Louis, MO, USA) was dissolved in assay buffer [35 mM Tris–HCl, 20 mM MgCl2, 2 mM CaCl2, 2.5 × SYBR Safe (Invitrogen), pH 7.8] at a concentration of 100 µg/mL. The solution was kept at 50 °C for ten minutes, then mixed 1:2 with 2% ultra-pure agarose (Invitrogen) and poured into plastic trays. After solidification, 2 µL sample or standard (Dornase alfa; Roche, Basel, Switzerland) were loaded into wells of 1 mm diameter. Gels were incubated for ten hours at 37 °C. At six and ten hours, remaining fluorescence was recorded with a Fusion FX imaging system (Vilber, Marne-la-Vallée, France). DNase activity of samples was compared to a six-point standard curve with a detection limit of 0.78 mU/mL.

### 2.4. Statistics

**Figure 2:**
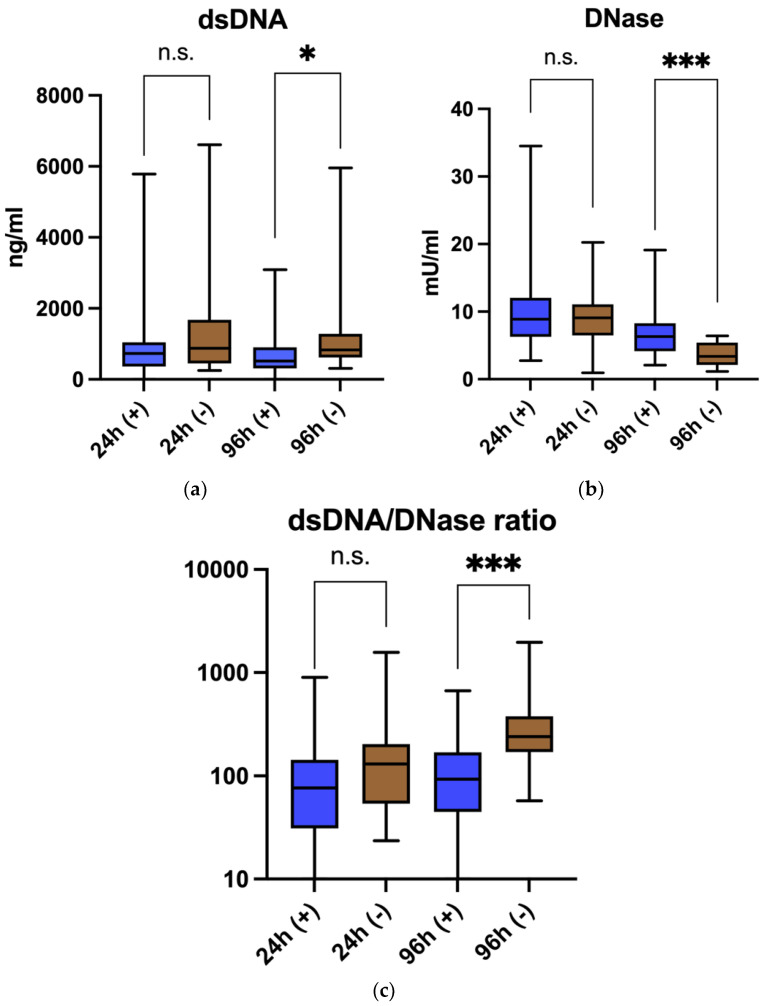
Boxplots comparing (**a**) dsDNA [ng/mL] after 24 and 96 h, (**b**) DNase [mU/mL] after 24 and 96 h, (**c**) dsDNA/DNase ratio after 24 and 96 h. Abbreviations: 24 h (+): value after 24 h in patients alive/CPC < 3 after six months; 24 h (−): value after 24 h in patients deceased/CPC > 2 after six months; 96 h (+): value after 96 h in patients alive/CPC < 3 after six months; 24 h (−): value after 96 h in patients deceased/CPC > 2 after six months; Whiskers represent minimum and maximum values. Asterisks indicate statistical significance (*: *p* < 0.05; ***: *p* < 0.001). For the y-axis of Figure 2c, a log10 scale was used for a more comprehensive visualization. Abbreviations: DNase: deoxyribonuclease; dsDNA: double-stranded DNA; n.s.: not specified.

Stata 16.1 (Stata Statistical Software: Release 16. StataCorp LLC, College Station, Texas, United States) was used for all statistical analyses. Continuous data were expressed as median ± interquartile range (IQR). Differences between independent groups were calculated using the Mann Whitney U test. Categorical data were expressed as numbers (percentage). Missing data were handled using listwise deletion. A Chi-square test was used to calculate univariate differences between groups. A combined endpoint consisting of death or unfavorable neurological outcome (CPC ≥ 3) after six months was defined. Based on the published study of Ondracek et al., an optimal cutoff value was calculated using the Youden index [12]. Receiver operating curve (ROC) analysis was performed, and the area under the curve (AUC) was calculated. Associations of the dsDNA/DNase ratio as a continuous and as a binary variable (above and below the cutoff value) were evaluated using logistic regression analysis. A univariable as well as a multivariable model that adjusted for confounding factors (age, sex, sequential organ failure assessment (SOFA) score, and initial rhythm) were created. Calculated odds ratios (OR) and adjusted odds ratios (aOR) with respective 95% confidence intervals (95% CI) were used. The selection of confounders was based on previously published and established variables. All tests were two-sided and a *p*-value < 0.05 was considered statistically significant. Figure 2a–c were generated using GraphPad Prism (GraphPad Software, San Diego, CA, USA). Data are presented as box plots, with whiskers defined according to minimum and maximum range.

## 3. Results

**Figure A2:**
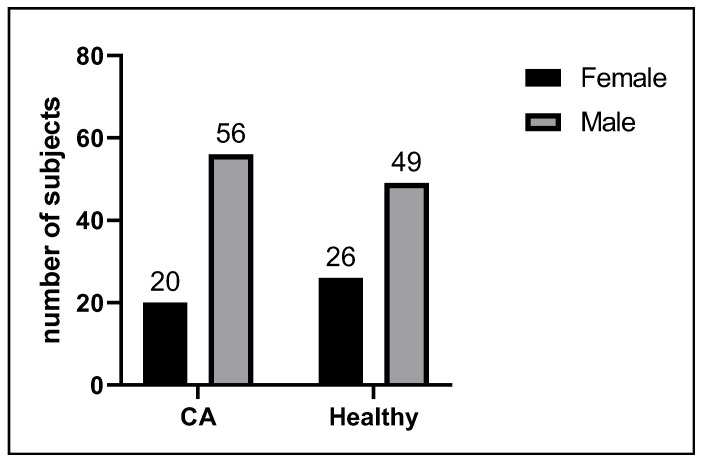
Sex distribution. Abbreviations: CA: cardiac arrest.

**Figure A3:**
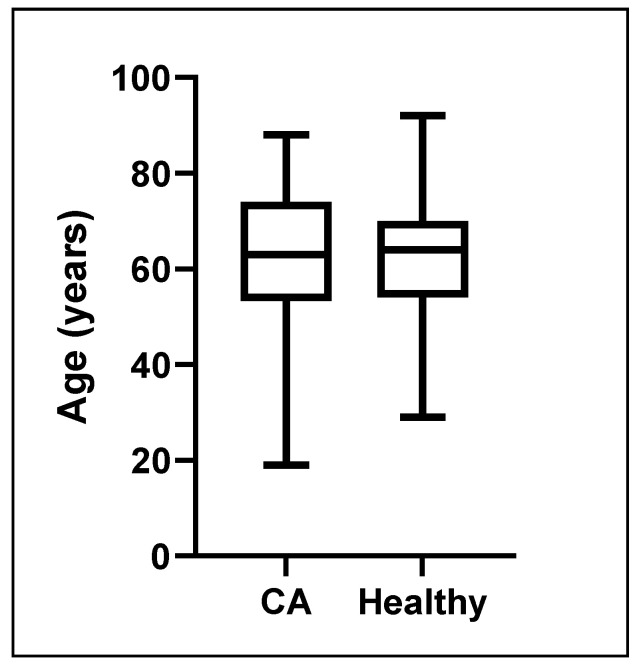
Age distribution. Abbreviations: CA: cardiac arrest.

A total of 76 patients were included in the final analysis of whom 19 met the primary endpoint. No patients were lost in follow-up. Table 1 shows baseline characteristics, CPR-specific data, intensive care measures, and outcome data in patients with favorable vs. non-favorable outcomes. The median dsDNA levels 24 h after admission were 727.7 (364.8–1012.5) ng/mL and decreased to 518.6 (312.2–877.9) ng/mL at 96 h in survivors with good neurologic outcome. In patients who met the primary endpoint, the median dsDNA level was 878.8 (449.3–1674.6) ng/mL at 24 h and 830.0 (614.4–1281.9) ng/mL at 96 h. As for DNase, in patients with favorable outcome, median levels after 24 h were 8.9 (6.4–11.8) mU/mL, and 6.3 (4.2–8.1) mU/mL after 96 h respectively. DNase levels in patients with unfavorable outcome were 9.1 (6.5–11.1) mU/mL at 24 h and 3.4 (2.1–5.4) mU/mL at 96 h. The corresponding ratios could be calculated at 24 h as 76.7 (31.3–142.0) for good outcome and 130.9 (53.9–203.3, *p* = 0.059) for unfavorable outcome. At 96 h, the ratio was 93.1 (44.6–166.4) for good outcome and 239.9 (175.6–355.9, *p* < 0.001) in patients with unfavorable outcome. These results are provided in Figure 2. Furthermore, Table 2 provides a summary of all obtained laboratory data in patients with favorable vs. non-favorable outcomes. For the control group seventy-five healthy subjects were included, of whom 49 (65.3%) were male (see Figure A2), and the median age was 64 (IQR 54-70) years (see Figure A3). Compared to healthy individuals, patients 96 h after CA had significantly higher dsDNA levels (mean 848.3 ± 880.9 ng/mL vs. 275.7 ± 78.3 ng/mL, *p* < 0.0001), lower DNase levels (6.0 ± 3.2 mU/mL vs. 7.0 ± 2.8 mU/mL, *p* = 0.047), and correspondingly higher dsDNA/DNase ratios (202.5 ± 283.7 vs. 45.3 ± 21.4, *p* < 0.0001). Table A1 provides an overview of the group of Healthy controls.

**Figure 3:**
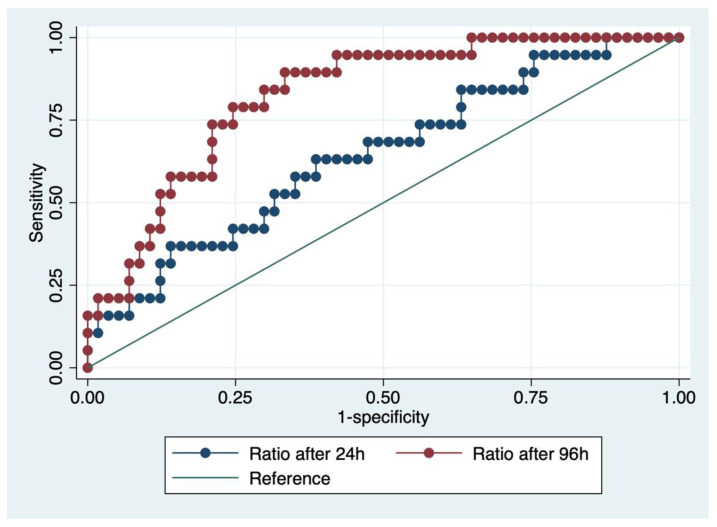
Receiver-operating-characteristics (ROC) curves for dsDNA/DNase ratio after 24 and 96 h.

Using ROC analysis, an area under the curve (AUC) of 0.65 (95% CI 0.50–0.79) was found for dsDNA/DNase after 24 h versus 0.83 (95% CI 0.73–0.92) after 96 h (*p* = 0.03). Corresponding ROC curves are shown in Figure 3. The empirical cut-off for dsDNA/DNase after 96 h was determined to be 149.97 using the Youden index. The area under the curve (AUC) for dsDNA/DNase ratio above the cut-off was 0.78. Due to better performance, only the dsDNA/DNase ratio after 96 h was used for subsequent analyses. Patients were then divided into two groups (below and above cut-off) according to dsDNA/DNase ratio 96 h after admission. Baseline characteristics were similar between both groups with a dsDNA/DNase ratio after 96 h below and above the empirical cut-off (median age 62.5 vs. 64.5 years, *p* = 0.30; female sex 27.5 vs. 25.0%, *p* = 0.80; median body mass index 26.2 vs. 25.7 kg/m^2^, *p* = 0.99; OHCA 80.0 vs. 91.7%, *p* = 0.15). There were also no significant differences regarding primary rhythm (ventricular fibrillation 84.6 vs. 74.3%, asystole 5.1 vs. 20.0%, pulseless electrical activity 7.7 vs. 5.7%, unknown rhythm 2.6 vs. 0%, *p* = 0.20), rates of bystander CPR (60.7 vs. 56.0%, *p* = 0.16), cause of arrest (acute coronary syndrome 66.7 vs. 71.4%, pulmonary embolism 5.1 vs. 8.6%, primary rhythm event 23.1 vs. 11.4%, asphyxia 5.1 vs. 5.7%, *p* = 0.68), and comorbidities (see Table 3). Regarding intensive care measures, patients with a ratio above the cut-off received targeted temperature management more often (86.1 vs. 62.5%, *p* = 0.020). No significant difference but a trend toward more invasive measures was observed for mechanical ventilation (100 vs. 90%, *p* = 0.051), renal replacement therapy (13.9 vs. 2.5%, *p* = 0.066), and blood transfusions (19.4 vs. 5%, *p* = 0.052). No significant difference was found for use of vasopressors (97.2 vs. 87.5%, *p* = 0.12). Longer durations of mechanical ventilation (median 217.6 vs. 56.8 h, *p* = 0.007) and intensive care stays (median 291.3 vs. 157.6 h, *p* = 0.020) were also observed in this group. Disease severity, reflected by SOFA (10 vs. 11 pts., *p* = 0.009) and SAPS II scores (median 75.5 vs. 80.5 pts., *p* = 0.015), was higher in the group with a dsDNA/DNase ratio above the cut-off. For detailed results regarding baseline characteristics, CPR-specific data, intensive care measures and outcome data, see Table 3. With regard to laboratory values, standard laboratory tests showed no statistically significant differences between the two groups. MCP-1 levels were significantly higher at 24 h (median 343.8 vs. 818.8 pg/mL, *p* = 0.007), which resolved at 96 h (130.1 vs. 170.8 pg/mL, *p* = 0.066) after CA in individuals with dsDNA/DNase ratios above the empirical cut-off. IL-8 levels were overall low at 24 h (median 14.6 vs. 25.6 pg/mL, *p* = 0.056) and mostly below the detection limit of the assay at 96 h (9.1 vs. 14.9 pg/mL, *p* = 0.069). Detailed laboratory results are shown in Table 4.

**Figure 4:**
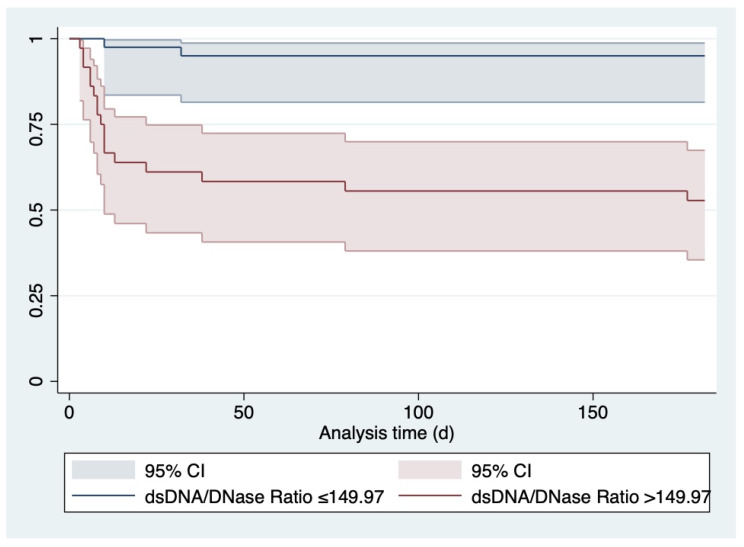
Kaplan–Meier plot for patients with dsDNA/DNase Ratio ≤ 149.97 versus dsDNA/DNase Ratio > 149.97. 96 h after ICU admission. Abbreviations: CI: confidence interval; ICU: intensive care unit.

Regarding the regression analysis, an elevated dsDNA/DNase ratio after 96 h was associated with an unfavorable outcome after six months both as a continuous (aOR 1.006, 95% CI 1.0017–1.0094, *p* = 0.005), as well as a binary (above median aOR 17.000, 95% CI 3.5537–81.3248, *p* < 0.001) variable. In the multivariable model, the association of both dsDNA/DNase ratio as a continuous variable (aOR 1.004, 95% CI 1.0004–1.0079, *p* = 0.029) and as a binary variable (aOR 11.551, 95% CI 2.2239–59.9926, *p* = 0.004) remained independently predictive of the outcome after adjustment for potential confounders. The corresponding Kaplan–Meier curve is given in Figure 4.

## 4. Discussion

This study demonstrates good performance of dsDNA/DNase ratio 96 h after cardiac arrest as a marker for prediction of medium-term outcome after cardiac arrest. We thus may provide another piece in the puzzle for ongoing inflammation as a negative outcome predictor in post-CA patients.

Evidence on the role of cell-free DNA and/or NET formation after CA is scarce. Arnalich et al. measured the amount of cell-free DNA in plasma within two hours of CA in 85 OHCA survivors by real-time quantitative PCR assay for β-globin. They showed that the amount of cell-free DNA is a good predictor of short-term outcome (24-h and in-hospital mortality) after successful resuscitation [16]. Mauracher et al. studied the levels of citrullinated histone H3, cell-free DNA, and nucleosomes at baseline and twelve hours after admission in 62 CA patients. They were able to show an association of these biomarkers at an early time point with poor neurological 30-day-outcome [13]. Ondracek et al. recently demonstrated an association between the ratio of dsDNA levels and DNase activity 24 h after the return of spontaneous circulation and 30-day mortality in 64 OHCA survivors. They observed significantly decreasing dsDNA/DNase ratios immediately after the acute phase (24 h) [12]. In an attempt to confirm and extend these previous findings in our center, a cohort of CA patients admitted to the ICU was investigated adding a follow-up measurement at 96 h.

In general, it is not possible with our work to directly prove that NET formation itself played a relevant role, because only cell-free DNA and DNase were measured. However, it is to date not possible to accurately quantify NETs or NET formation in vivo other than by using differentially disputed surrogate markers. In favor of dsDNA as adequate marker, Mangold and colleagues present data of histologically quantified NETs in coronary thrombectomies from patients with ST-segment elevation myocardial infarction (STEMI), which correlated significantly with plasma dsDNA levels. Additionally, coronary NET burden correlated positively with electrocardiographic infarct resolution, infarct size, and negatively with DNase activity [17]. Moreover, non-degraded DNA in circulation appears to contribute to consecutive microvascular obstruction, which is an important feature of PCAS [18]. Although dsDNA is generally regarded as a surrogate marker for NET formation [19], the pathophysiological circumstances might strongly determine the probability that cell-free DNA actually originates from NETing neutrophils, as indicated in STEMI. In the present study, about 68% of patients suffered a CA because of ACS, increasing the likelihood of neutrophil activation in these patients.

Multiple novel biomarkers have been studied in the past in patients after cardiac arrest [6,7,20]. Moreover, classical indicators of inflammation such as leukocyte and neutrophil counts were associated with a worse medium- to long-term outcome after resuscitation [21]. These findings certainly support the hypothesis that neutrophils influence the pathogenesis of PCAS. Furthermore, a trend toward higher IL-8 levels both, at 24 h and 96 h, was observed in patients with higher dsDNA/DNase ratio, implicating the recruitment of neutrophils. In general, exorbitant and/or persistent inflammation after CA is associated with a negative outcome [22]. Moreover, most pro-inflammatory cytokines and chemokines show temporal dynamics within the first 72 h after CA, whereas various standard interventions, such as targeted temperature management, have been observed to affect these biomarker levels [6,20,23]. The pro-inflammatory chemokines interleukin-8 and MCP-1 are certainly among the most studied novel biomarkers [6,7]. Regarding “classical inflammatory markers” in our study, a difference was only shown for C-reactive protein but not leukocyte counts. Yet, markedly higher levels of IL-8 at 24 h and 96 h were observed in individuals with unfavorable outcome, whereas no difference was shown for MCP-1. Only when comparing patients with a dsDNA/DNase ratio above and below the empirical cut-off, significantly higher MCP-1 levels could be shown in the group with higher dsDNA burden after 24 h, whereas only a trend remained after 96 h. MCP-1 functions chemotactically and recruits monocytes to the affected tissue during inflammatory processes. Interestingly, Cheng et al. demonstrated an association of higher MCP-1 levels and lower 30-day mortality in a cohort of cardiogenic shock patients after acute myocardial infarction [24]. Moreover, in their work, biomarker levels increased within the first three days, whereas a decreasing trend was observed in our study. However, direct comparison might not be appropriate considering that the observed MCP-1 levels in our patients were higher by a multiple from the beginning, which is also consistent with the findings of Bisschops et al. suggesting a stronger inflammatory component in PCAS compared with cardiogenic shock [6]. Interestingly, MCP-1 was recently shown to prime neutrophils for NET formation in a cohort of STEMI patients [25], highlighting a potential connection between both parameters also in CA patients. As mentioned above, balanced inflammation is certainly crucial for a beneficial outcome, which may also explain the different findings of various authors regarding the effect of elevated MCP-1 levels [24,26]. Importantly, patients with unfavorable outcome showed decreased DNase activity 96 h after ICU admission, suggesting that a decreased repair capacity might play a role in patients with poor outcome, in addition to increased inflammation and cell death. Timely degradation of circulating DNA-histone-complexes is a prerequisite to prevent pro-inflammatory signaling [9,10] and, in the long-term, autoimmune phenomena [27]. Overall, higher DNase activity seems to represent a survival benefit as a homozygous mutation in DNase1 was independently associated with cardiovascular and all-cause mortality after STEMI [11]. This combined marker could be useful in diseases with a significant inflammatory component or sequelae due to vascular obstruction. Data on the accumulation of NETs and dsDNA have been published for myocardial infarction, ischemic stroke, pulmonary embolism, chronic thromboembolic pulmonary hypertension, or coronavirus disease 19 (COVID-19) [11,28,29,30,31]. Other more specific approaches also use the detection of circulating cell-free DNA, for example, for the early diagnosis of cancer [32]. Consideration of the influence of DNase activity in different disease entities could significantly improve predictive power. Accordingly, DNase has been suggested as therapeutic intervention to improve patient outcome in several diseases [33,34], and indeed multiple studies exploring the benefits of DNA degradation are currently registered.

From a scientific perspective, it would be important to investigate the exact role of NETs in PCAS. This would eventually require animal models and/or post-mortem histological studies of relevant human organ systems. Yet, from an intensivist’s perspective, it is crucial to find easily determinable markers that are prognostically relevant and meaningfully affect clinical patient management. Biomarkers that can be measured in peripheral blood are suitable for this purpose, with their dynamics being particularly relevant. Accordingly, in addition to dsDNA, and the capacity for degradation by DNase, other (complementary) inflammatory markers (IL-8, MCP-1) were also examined. Whereas IL-8 and MCP-1 levels were decreasing over time, the limited degradation capacity of patients meeting the primary endpoint seemed to cause accumulation of circulating dsDNA as represented by the ratio to DNase activity. Extending the observation time to 96 h specifically improved the level of prediction as compared to previous studies [12]. However, the present analysis could only include patients who have not immediately succumbed to their condition, rather focusing on the hazards of inflammatory sequelae. Whether and at what point in time the determination of various novel biomarkers in patients after cardiac arrest plays a relevant prognostic and possibly therapeutic role in the distant future remains to be confirmed. Nevertheless, the inflammatory pillar of PCAS certainly plays a highly relevant role in the pathogenesis and especially burden of morbidity and mortality.

### Limitations

One main limitation of our study is small sample size. Accordingly, the event rate is also low, especially with regard to poor neurological outcome. Therefore, the number of individuals who reached the primary endpoint is low. Unfortunately, few preclinical data were available, such as time-to-CPR, time-to-ROSC, number of shocks, and cumulative doses of epinephrine. Nevertheless, we believe that the very well-characterized patient population supports the value of this work and also the comparatively late time point of the second laboratory sampling could provide the basis for future studies.

## 5. Conclusions

This observational single center study shows a strong prognostic performance for cell-free dsDNA/DNase ratio at 96 h in patients after primary survived cardiac arrest. Whether this reflects primarily enhanced cell death, persistent and generalized inflammation, or impaired dsDNA degradation, remains to be determined by future research in large-scale studies with extensive cytological and histologic work-up.

## Figures and Tables

**Table 1:** Baseline characteristics, CPR-specific data, intensive care measures, and outcome data in patients with favorable vs. unfavorable outcome (death or CPC ≥ 3 after six months).

	Favorable Outcome at Six Months (*n* = 57)	Unfavorable Outcome at Six Months (*n* = 19)	*p*-Value
Age—median yrs. (IQR)	62.0 (54.0–71.0)	65.0 (53.0–77.0)	0.41
Female sex—*n* (%)	24.6 (14.0)	31.6 (6.0)	0.55
BMI—median kg/m^2^ (IQR)	26.2 (24.5–29.2)	26.1 (24.5–26.2)	0.53
OHCA—*n* (%)	48 (84.2)	17 (89.5)	0.57
Bystander CPR—*n* (%)	-	-	0.45
Yes	23 (60.5)	8 (53.3)	-
No	6 (15.8)	5 (33.3)	-
In-hospital	7 (18.4)	2 (13.3)	-
CA during transport	2 (5.3)	0 (0.0)	-
Initial rhythm—*n* (%)	-	-	0.026
Ventricular fibrillation	47 (85.5)	12 (63.2)	-
Asystole	3 (5.5)	6 (31.6)	-
Pulseless electrical activity	4 (7.3)	1 (5.3)	-
Unknown	1 (1.8)	0 (0.0)	-
Cause of arrest—*n* (%)	-	-	0.17
ACS	39 (69.6)	12 (66.7)	-
Pulmonary embolism	3 (5.4)	2 (11.1)	-
Primary rhythm event	12 (21.4)	1 (5.6)	-
Asphyxia	2 (3.6)	2 (11.1)	-
Unknown	0 (0.0)	1 (5.6)	-
Comorbidities—*n* (%)	-	-	-
Arterial hypertension	46 (85.2)	8 (80.0)	0.68
Diabetes mellitus	12 (21.4)	5 (31.3)	0.41
Hyperlipidemia *	38 (66.7)	8 (42.1)	0.058
Smoking history	27 (58.7)	8 (72.7)	0.39
CKD	10 (17.5)	4 (22.2)	0.66
COPD	7 (12.3)	2 (10.5)	0.84
Intensive care measures—*n* (%)	-	-	-
Mechanical ventilation	53 (93.0)	19 (100)	0.24
Targeted temperature management	40 (70.2)	16 (84.2)	0.23
Use of vasopressors	51 (89.5)	19 (100.0)	0.14
RRT	3 (5.3)	3 (15.8)	0.14
Antibiotic therapy	51 (89.5)	19 (100.0)	0.14
RBC transfusions	4 (7.0)	5 (26.3)	0.024
Coronary angiography—*n* (%)	50 (87.7)	13 (68.4)	0.053
PCI—*n* (%)	36 (63.1)	11 (57.9)	0.68
SOFA-Score—median pts. (IQR)	10 (8–12)	12 (10–12)	0.013
SAPS II—median pts. (IQR)	76 (70–81)	84 (76–87)	0.004
Duration mechanical ventilation—median hrs. (IQR)	41.5 (24.5–61.5)	190.0 (136.5–322.0)	<0.001
Duration ICU stay—median hrs. (IQR)	119.5 (86.0–209.5)	233.0 (136.5–322.0)	0.007
Survival > 6 months—*n* (%)	57 (100.0)	0 (0.0)	<0.001

Abbreviations: ACS: acute coronary syndrome; BMI: body mass index; CA: cardiac arrest; CKD: chronic kidney disease; COPD: chronic obstructive pulmonary disease; CPR: cardiopulmonary resuscitation; DNase: deoxyribonuclease; dsDNA double-stranded DNA; hrs.: hours; ICU: intensive care unit; LDL: low density lipoprotein; IQR: interquartile range; OHCA: out of hospital cardiac arrest; PCI: percutaneous coronary intervention; pts.: points; RBC: red blood cell; RRT: renal replacement therapy; SAPS II: Simplified Acute Physiology Score; SOFA: sequential organ failure assessment; yrs.: years; * LDL > 100 mg/dL or documented.

**Table 2:** Laboratory data in patients with favorable vs. unfavorable outcome (death or CPC ≥ 3 after six months).

	Favorable Outcome at Six Months (*n* = 57)	Unfavorable Outcome at Six Months (*n* = 19)	*p*-Value
Initial pH median (IQR)	7.26 (7.13–7.33)	7.15 (7.02–7.25)	0.058
Initial lactate—mmol/L median (IQR)	3.7 (2.2–5.9)	6.1 (2.9–10.0)	0.037
pO_2_—mmHg median (IQR)	110.2 (81.3–184.2)	129.5 (98.5–213.6)	0.23
pCO_2_—mmHg median (IQR)	40.5 (35.8–51.6)	45.3 (39.9–53.0)	0.21
Hemoglobin—g/dL median (IQR)	14.2 (13.1–15.2)	13.5 (12.3–14.7)	0.14
Leukocytes—G/L median (IQR)	14.2 (10.4–18.3)	14.9 (11.9–17.3)	0.71
Thrombocytes—G/L median (IQR)	231.0 (190.0–274.0)	230.0 (170.0–311.0)	0.88
CRP—mg/dL median (IQR)	0.3 (0.1–0.6)	0.5 (0.2–1.6)	0.039
IL-8 24 h—pg/mL median (IQR)	17.3 (9.4–30.5)	30.8 (19.6–69.8)	0.018
IL-8 96 h—pg/mL median (IQR)	9.4 (4.8–16.8)	20.9 (14.8–24.5)	<0.001
MCP-1 24 h—pg/mL median (IQR)	504.1 (218.7–875.6)	495.2 (273.6–1006.2)	0.55
MCP-1 96 h—pg/mL median (IQR)	145.6 (86.2–218.7)	163.5 (97.1–340.0)	0.46
GGT—U/L median (IQR)	58.0 (32.0–94.5)	61.0 (38.0–98.0)	0.83
AST—U/L median (IQR)	184.0 (98.0–287.0)	191.0 (105.0–395.0)	0.62
ALT—U/L median (IQR)	134.5 (71.0–259.0)	200.0 (110.0–263.0)	0.25
Bilirubin—mg/dl median (IQR)	0.5 (0.4–0.6)	0.5 (0.3–0.6)	0.78
Creatinkinase—U/L median (IQR)	215.0 (142.0–625.0)	241.0 (185.0–474.0)	0.47
Troponin T—ng/L median (IQR)	113.0 (41.0–447.0)	147.0 (65.0–197.0)	0.67
TSH—mU/L median (IQR)	2.7 (1.4–3.8)	2.5 (1.0–3.8)	0.96
Hba_1c_—% median (IQR)	5.5 (5.2–5.8)	5.6 (5.3–6.1)	0.24
LDL—mg/dL median (IQR)	80.0 (52.0–107.0)	66.5 (48.0–119.0)	0.71
dsDNA 24 h—ng/mL median (IQR)	727.7 (364.8–1012.5)	878.8 (449.3–1674.6)	0.11
dsDNA 96 h—ng/mL median (IQR)	518.6 (312.2–877.9)	830.0 (614.4–1281.9)	0.015
DNase 24 h—mU/mL median (IQR)	8.9 (6.4–11.8)	9.1 (6.5–11.1)	0.69
DNase 96 h—mU/mL median (IQR)	6.3 (4.2–8.1)	3.4 (2.1–5.4)	<0.001
dsDNA/DNase Ratio 24 h median (IQR)	76.7 (31.3–142.0)	130.9 (53.9–203.3)	0.059
dsDNA/DNase Ratio 96 h median (IQR)	93.1 (44.6–166.4)	239.9 (175.6–355.9)	<0.001

Abbreviations: ALT: Alanine transaminase; AST: Aspartate transaminase; CRP: C-reactive protein; DNase: deoxyribonuclease; dsDNA double-stranded DNA; GGT: gamma-glutamyltransferase; Hba_1c_: glycohemoglobin; IL: interleukin; IQR: interquartile range; LDL: low density lipoprotein; paCO_2_: arterial partial pressure of carbon dioxide; paO_2_: arterial partial pressure of oxygen; TSH: thyrotropin.

**Table 3:** Baseline characteristics, CPR-specific data, intensive care measures, and outcome data according to dsDNA/DNase ratio below and above the empirical cut-off value.

	dsDNA/DNase Ratio ≤149.97 (*n* = 40)	dsDNA/DNase Ratio >149.97 (*n* = 36)	*p*-Value
Age—median yrs. (IQR)	62.5 (53.0–72.0)	64.5 (56.0–75.5)	0.30
Female sex—*n* (%)	11 (27.5)	9 (25.0)	0.80
BMI—median kg/m^2^ (IQR)	26.2 (24.5–28.7)	25.7 (24.5–29.3)	0.99
OHCA—*n* (%)	32 (80.0)	33 (91.7)	0.15
Bystander CPR—*n* (%)	-	-	0.16
Yes	17 (60.7)	14 (56.0)	-
No	3 (10.7)	8 (32.0)	-
In-hospital	7 (25.0)	2 (8.0)	-
CA during transport	1 (3.6)	1 (4.0)	-
Initial rhythm—*n* (%)	-	-	0.20
Ventricular fibrillation	33 (84.6)	26 (74.3)	-
Asystole	2 (5.1)	7 (20.0)	-
Pulseless electrical activity	3 (7.7)	2 (5.7)	-
Unknown	1 (2.6)	0 (0.0)	-
Cause of arrest—*n* (%)	-	-	0.68
ACS	26 (66.7)	25 (71.4)	-
Pulmonary embolism	2 (5.1)	3 (8.6)	-
Primary rhythm event	9 (23.1)	4 (11.4)	-
Asphyxia	2 (5.1)	2 (5.7)	-
Unknown	0 (0.0)	1 (2.9)	-
Comorbidities—*n* (%)	-	-	-
Arterial hypertension	33 (86.8)	21 (80.8)	0.51
Diabetes mellitus	8 (20.0)	9 (28.1)	0.42
Hyperlipidemia *	27 (67.5)	19 (52.8)	0.19
Smoking history	21 (63.6)	14 (58.3)	0.68
CKD	8 (20.0)	6 (17.1)	0.75
COPD	5 (12.5)	4 (11.1)	0.85
Intensive care measures—*n* (%)	-	-	-
Mechanical ventilation	36 (90.0)	36 (100.0)	0.051
Targeted temperature management	25 (62.5)	31 (86.1)	0.020
Use of vasopressors	35 (87.5)	35 (97.2)	0.12
RRT	1 (2.5)	5 (13.9)	0.066
Antibiotic therapy	35 (87.5)	35 (97.2)	0.12
RBC transfusions	2 (5.0)	7 (19.4)	0.052
Coronary angiography—*n* (%)	34 (85.0)	29 (80.6)	0.61
PCI—*n* (%)	24 (60.0)	23 (63.9)	0.73
SOFA-Score—median pts. (IQR)	10 (8–11.5)	11 (10–12)	0.009
SAPS II—median pts. (IQR)	75.5 (70–81)	80.5 (75–85.5)	0.015
Duration mechanical ventilation—median hrs. (IQR)	36.2 (20.8–48.5)	112.5 (46.2–213.0)	<0.001
Duration of ICU stay—median hrs. (IQR)	113.8 (71.0–198.5)	187.5 (116.3–315.5)	0.008
Survival >6 months—*n* (%)	38 (95.0)	19 (52.8)	<0.001

Abbreviations: ACS: acute coronary syndrome; BMI: body mass index; CA: cardiac arrest; CKD: chronic kidney disease; COPD: chronic obstructive pulmonary disease; CPR: cardiopulmonary resuscitation; DNase: deoxyribonuclease; dsDNA double-stranded DNA; hrs.: hours; ICU: intensive care unit; LDL: low density lipoprotein; IQR: interquartile range; OHCA: out of hospital cardiac arrest; PCI: percutaneous coronary intervention; pts.: points; RBC: red blood cell; RRT: renal replacement therapy; SAPS II: Simplified Acute Physiology Score; SOFA: sequential organ failure assessment; yrs.: years; * LDL > 100 mg/dl or documented.

**Table 4:** Laboratory data according to dsDNA/DNase ratio below and above the empirical cut-off value.

	dsDNA/DNase Ratio ≤149.97 (*n* = 40)	dsDNA/DNase Ratio >149.97 (*n* = 36)	*p*-Value
Initial pH median (IQR)	7.28 (7.13–7.35)	7.22 (7.08–7.28)	0.066
Initial lactate—mmol/L median (IQR)	3.9 (2.3–6.2)	4.7 (2.4–8.5)	0.42
pO_2_—mmHg median (IQR)	142.0 (86.3–220.2)	107.4 (84.1–150.6)	0.20
pCO_2_—mmHg median (IQR)	39.8 (36.6–51.6)	43.2 (38.6–51.3)	0.36
Hemoglobin—g/dL median (IQR)	13.8 (12.9–15.2)	14.4 (13.3–14.8)	0.40
Leukocytes—G/L median (IQR)	14.5 (10.4–17.0)	14.2 (11.7–19.0)	0.44
Thrombocytes—G/L median (IQR)	238.0 (188.5–274.0)	228.5 (199.0–299.5)	0.80
CRP—mg/dL median (IQR)	0.3 (0.1–0.9)	0.4 (0.2–0.8)	0.58
IL-8 24 h—pg/mL median (IQR)	14.6 (9.1–26.1)	25.6 (14.6–51.7)	0.056
IL-8 96 h—pg/mL median (IQR)	9.1 (5.9–21.0)	14.9 (9.4–24.0)	0.069
MCP-1 24 h—pg/mL median (IQR)	343.8 (152.2–723.2)	818.8 (361.3–1053.6)	0.007
MCP-1 96 h—pg/mL median (IQR)	130.1 (78.5–206.7)	170.8 (97.5–334.6)	0.066
GGT—U/L median (IQR)	58.0 (33.0–104.0)	56.0 (31.0–95.0)	0.68
AST—U/L median (IQR)	195.5 (95.0–394.5)	190.0 (107.0–287.0)	0.80
ALT—U/L median (IQR)	165.0 (62.0–257.0)	128.0 (101.0–263.0)	0.58
Bilirubin—mg/dL median (IQR)	0.5 (0.4–0.7)	0.5 (0.3–0.6)	0.26
Creatinkinase—U/L median (IQR)	223.0 (149.0–502.5)	229.0 (151.0–700.5)	0.55
Troponin T—ng/L median (IQR)	125.5 (41.0–334.0)	128.0 (54.0–573.0)	0.81
TSH—mU/L median (IQR)	3.0 (1.7–3.9)	2.1 (1.0–3.5)	0.12
Hba_1c_—% median (IQR)	5.5 (5.2–5.8)	5.5 (5.3–5.9)	0.96
LDL—mg/dl median (IQR)	71.5 (45.0–101.0)	85.0 (53.0–123.0)	0.16
dsDNA 24 h—ng/mL median (IQR)	489.1 (352.5–872.6)	977.3 (676.6–1586.9)	<0.001
dsDNA 96 h—ng/mL median (IQR)	390.3 (259.7–533.3)	1006.5 (706.1–1460.3)	<0.001
DNase 24 h—mU/mL median (IQR)	8.9 (4.8–11.7)	9.0 (7.2–11.6)	0.53
DNase 96 h—mU/mL median (IQR)	7.2 (5.5–8.9)	3.9 (3.3–5.1)	<0.001
dsDNA/DNase Ratio 24 h median (IQR)	63.1 (28.3–137.2)	103.6 (58.6–211.7)	0.006
dsDNA/DNase Ratio 96 h median (IQR)	56.1 (32.5–93.1)	236.8 (177.9–343.9)	<0.001

Abbreviations: ALT: Alanine transaminase; AST: Aspartate transaminase; CRP: C-reactive protein; DNase: deoxyribonuclease; dsDNA double-stranded DNA; GGT: gamma-glutamyltransferase; Hba_1c_: glycohemoglobin; IL: interleukin; IQR: interquartile range; LDL: low density lipoprotein; paCO_2_: arterial partial pressure of carbon dioxide; paO_2_: arterial partial pressure of oxygen; TSH: thyrotropin.

## Data Availability

All data relevant for this study will be given by the authors upon specific request. Patients or the public were not involved in the design, or conduct, or reporting, or dissemination plans of our research.

## Appendix A

**Table A1:** Healthy controls. Abbreviations: yrs: years.

		Age—yrs	DNase—mU/mL	dsDNA—ng/mL	dsDNA/DNase Ratio
	Valid	75	72	75	72
	Missing	0	3	0	3
Mean		61.77	7.00	275.74	45.31
Standard error (mean)		1.26	0.33	9.04	2.52
Median		64.00	6.61	264.00	41.77
Standard deviation		10.92	2.82	78.31	21.41
Variance		119.20	7.95	6132.24	458.23
Minimum		29.00	2.93	150.99	14.69
Maximum		92.00	15.17	538.00	111.30
Percentile	25	54.00	4.60	216.75	30.68
	50	64.00	6.61	264.00	41.77
	75	70.00	8.49	323.53	57.63
